# Effect of Co and Gd Additions on Microstructures and Properties of FeSiBAlNi High Entropy Alloys

**DOI:** 10.3390/e20070487

**Published:** 2018-06-22

**Authors:** Sicheng Zhai, Wen Wang, Juan Xu, Shuai Xu, Zitang Zhang, Yan Wang

**Affiliations:** School of Materials Science and Engineering, University of Jinan, Jinan 250022, China

**Keywords:** high entropy alloys, elemental addition, annealing treatment, magnetic property, microhardness

## Abstract

FeSiBAlNi (W5), FeSiBAlNiCo (W6-Co), and FeSiBAlNiGd (W6-Gd) high entropy alloys (HEAs) were prepared using a copper-mold casting method. Effects of Co and Gd additions combined with subsequent annealing on microstructures and magnetism were investigated. The as-cast W5 consists of BCC solid solution and FeSi-rich phase. The Gd addition induces the formation of body-centered cubic (BCC) and face-centered cubic (FCC) solid solutions for W6-Gd HEAs. Whereas, the as-cast W6-Co is composed of the FeSi-rich phase. During annealing, no new phases arise in the W6-Co HEA, indicating a good phase stability. The as-cast W5 has the highest hardness (1210 HV), which is mainly attributed to the strengthening effect of FeSi-rich phase evenly distributed in the solid solution matrix. The tested FeSiBAlNi-based HEAs possess soft magnetism. The saturated magnetization and remanence ratio of W6-Gd are distinctly enhanced from 10.93 emu/g to 62.78 emu/g and from 1.44% to 15.50% after the annealing treatment, respectively. The good magnetism of the as-annealed W6-Gd can be ascribed to the formation of Gd-oxides.

## 1. Introduction

Recently, a new concept was proposed for high entropy alloys (HEAs), which has aroused wide attention and interest [1,2,3]. Generally, HEAs with equiatomic or near-equiatomic alloying elements mainly consist of face-centered cubic (FCC), body-centered cubic (BCC), or hexagonal closed-packed (HCP) solid solutions, and some intermetallic or amorphous phases. Owing to the special phase structure, HEAs usually possess excellent mechanical properties [4,5] and corrosion resistance [6], especially magnetic properties [7,8,9]. Several studies have reported that additions of certain elements into HEAs could induce the transformation of crystalline structures and further affect the related properties of HEAs [9,10,11]. The addition of Al, Ga, and Sn to the CoFeMnNi HEA induced the phase transition from FCC to ordered BCC phases, and further led to the significant improvement of the saturation magnetization (M_s_) [9]. The microstructural evolution of (FeCoNiCrMn)_100-x_Al_x_ HEA system transformed from the initial single FCC structure to final single BCC structure as Al concentration increased from 0 to 20 at. % [10]. Both the tensile fracture and yield strength were enhanced with increasing Al concentration. The HEAs phases are metastable in thermodynamics, therefore, would transform to the stable microstructure after subsequent annealing, which obviously affects the properties of HEAs to some degree [3,8,12,13,14,15]. Annealing under a given condition can lead to the occurrence of phase transition from a FCC to BCC phase for FeCoNi(CuAl)_0.8_ HEAs, resulting in a substantial increase in the M_s_ from 78.9 Am^2^/kg to 93.1 Am^2^/kg [15]. 

Recently, our group has prepared a series of as-milled FeSiBAlNi-based HEA powders using a mechanical alloying (MA) process [11,14,16]; these displayed an interesting microstructural evolution and magnetic properties. In the present study, the equiatomic FeSiBAlNiM (M = Co, Gd) HEAs were fabricated by a copper-mold spray casting technique. The effects of Co and Gd additions and subsequent annealing treatment on the microstructures, microhardness, and magnetism of the FeSiBAlNi HEAs were systematically investigated.

## 2. Experimental

The ingots of FeSiBAlNi, FeSiBAlNiCo, and FeSiBAlNiGd HEAs (denoted as W5, W6-Co, and W6-Gd, respectively) were prepared by an arc melting technique. The melting of these ingots was repeated at least five times to ensure the composition homogeneity in a Ti-gettered high-purity argon atmosphere. Then the ingots were remelted and made into 8 mm diameter rods by copper mold spray casting in an argon atmosphere. Then, the rods were annealed at given temperatures for two hours and cooled inside the furnace in the argon atmosphere. The annealing temperatures were set as two segments denoted as T_I_ and T_II_ in a low and high temperature region, respectively. There are 600 and 1000 °C for W5 HEA, 600 and 1000 °C for W6-Co HEA, and 650 and 1050 °C for W6-Gd HEA. The relatively high annealing temperatures selected for W6-Gd are attributed to the higher melting point (T_m_) than that of the other two samples, as shown in the differential scanning calorimetry (DSC) curves. 

Microstructural characterization of the as-cast and as-annealed HEAs were conducted by X-ray diffraction (XRD, Rigaku D8 Advance, Bruker, Germany) using Cu Kα radiation, field emission scanning electron microscopy (FESEM, QUANTA FEG 250 operated at 15 kV, Japan) coupled with energy dispersive spectrometry (EDS). The working distance used in SEM measurements was less than 10 mm. The thermal properties were analyzed by differential scanning calorimetry (DSC, TGA/DSC1, Mettler-Toledo, Greifensee, Switzerland) used under a continuous flow (30 mL/min) high-purity argon atmosphere at a heating rate of 10 K/min scanned from room temperature to 1400 °C. Microhardness of the tested HEAs was determined by a Vickers hardness tester (HV-10B), with a load of 200 g and a duration time of 15 s. The HV measurement for every tested sample was repeated ten times in order to obtain the average values. The coercive force (H_c_), M_s_, and remanence ratio (M_r_/M_s_, M_r_: remanence) were determined by an alternating gradient magnetometer (AGM) at room temperature with a maximum applied field of 14000 Oe.

## 3. Results and Discussion

**Figure 1:**
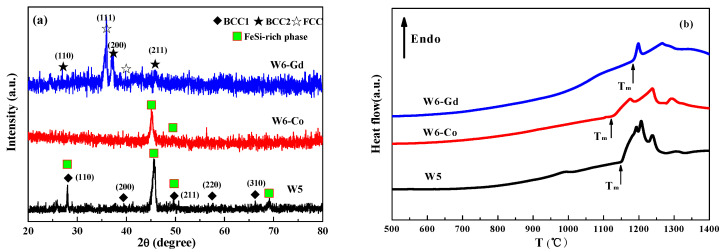
XRD patterns (**a**) and differential scanning calorimetry (DSC) curves (**b**) of the as-cast W5, W6-Co, and W6-Gd HEAs.

The XRD patterns of the as-cast W5, W6-Co, and W6-Gd HEAs are shown in Figure 1a. The as-cast W5 HEA consists of BCC1 (a = 4.475 Å) solid solution and FeSi-rich phase. The XRD pattern of the as-cast W6-Co HEA mainly displays the FeSi-rich phase of solution of other principal elements. In addition, other phase peaks may overlap with FeSi-rich phase peaks. Compared with the W5 and W6-Co HEAs, the effect of Gd addition on phase composition presents an obvious difference. The as-cast products of W6-Gd HEA exhibit the formation of new BCC2 (a = 4.484 Å) and FCC solid solutions. However, the FeSi-rich phase doesn’t appear. The phase products of the as-cast W5, W6-Co, and W6-Gd HEAs are presented in Table 1.

Figure 1b shows the DSC curves of the as-cast HEAs. The T_m_ value of W6-Co HEA is 1129 °C, which is lower than that of W5 (1152 °C). However, the Gd addition increases the value of T_m_, which reaches 1185 °C. 

**Figure 2:**
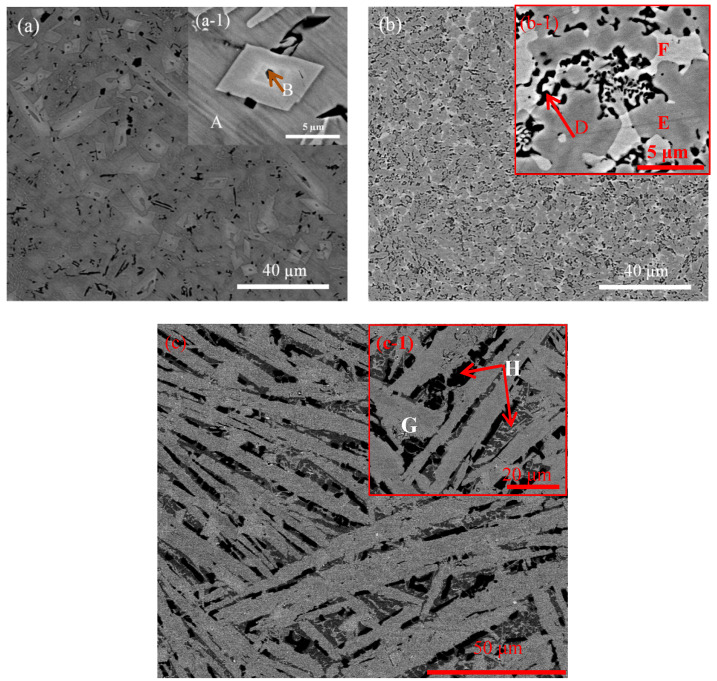
Field emission scanning electron microscopy (FESEM) micrographs of the as-cast high entropy alloys (HEAs): (**a**) W5; (**b**) W6-Co; and (**c**) W6-Gd. The insets of (a-1), (b-1), and (c-1) are the partial enlargements corresponding to (**a**), (**b**), and (**c**), respectively.

To further investigate the difference in morphologies and compositions caused by the Co and Gd additions, FESEM coupled with EDS analysis was carried out and is presented in Figure 2 and Table 2. As shown in Figure 2a, a larger number of polygonous light-grey phases distribute dispersedly in the matrix, as well as the irregular black phases with small size. The inset of Figure 2a-1 reveals one rhombic grain with edge sizes less than 8 μm. It needs to be noted that metalloid B as a light element can’t be accurately measured. Moreover, the B content of some samples is very small in most regions, therefore, they are omitted in the present study. According to the EDS results, matrix (A) contains more Al and Ni elements, and a certain amount of Fe and Si elements. Furthermore, there is a partial FeSi-rich phase in region (A) and the black region (B) is enriched with Fe and Si elements. This indicates that the FeSi-rich phase mainly exists in region (B), presenting an evenly distribution in the matrix. The rhombic grain (C) is mainly composed of Fe and B elements (instead, region (C) contains more B element above 10 at. %.). The Co addition induces the refinement of the precipitated grains (Figure 2b). The inset in Figure 2b-1 shows that the larger number of dark regions (D) with the smaller size exhibit a uniform distribution state and enrich Fe and Si elements. The gray region (E) is rich in the Ni element, and the bright-grey region (F) is poor in the Al element. Moreover, the component ratio of Fe and Si in regions (E) and (F) is close to 1:1. Although several phases appear in the SEM images, no peaks other than the FeSi-rich phase can be seen in the XRD results of as-cast W6-Co HEA (Figure 1). It suggests that the precipitates probably have a similar lattice constant and crystal structure concerning the matrix [17]. Moreover, it could be a complex compositional fluctuation in the as-cast W6-Co HEA [18]. Figure 2c and inset (Figure 2c-1) present that the as-cast W6-Gd HEA consists of coarse rod-like dendrites as the FCC-matrix phase. They are rich in each principal element with near-equiatomic ratio except for Al element (region (G)). However, the Al element segregates in the interdendritic grains ((H): dark- and deep-grey regions) corresponding to the BCC2 phase with less contents. Usually the precipitation pathways in HEAs can be very complex, and it is a particularly challenging topic, which remains to be studied [19]. 

**Figure 3:**
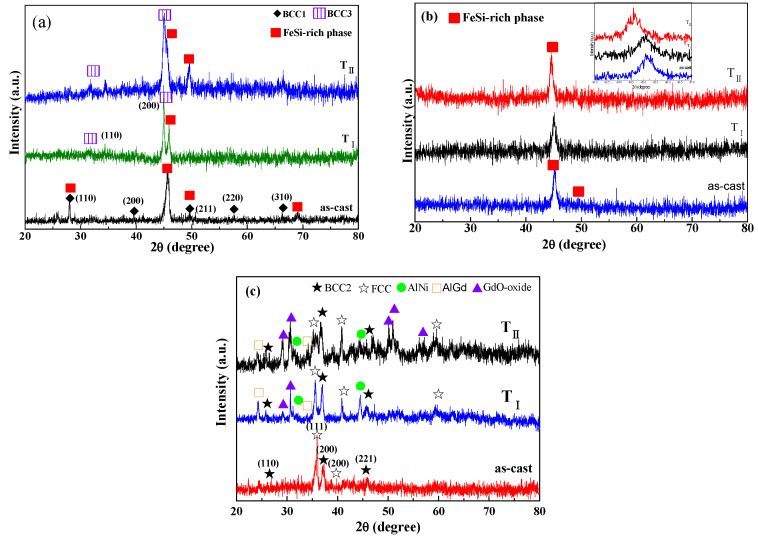
XRD patterns of the as-annealed HEAs at different temperatures (T_I_ and T_II_): (**a**) W5, (**b**) W6-Co, and (**c**) W6-Gd.

Figure 3 shows the XRD patterns of the as-annealed W5, W6-Co, and W6-Gd HEAs at different temperatures; their annealing products are also listed in Table 1. After annealing at T_I_, the annealed products of W5 HEAs consist of a new BCC3 (a = 4.033 Å) solid solution with a FeSi-rich phase. However, the contents of the FeSi-rich phase obviously decrease compared to that of the as-cast state. Moreover, the BCC1 solid solution disappears (Figure 3a). Via annealing at T_II_ the two obtained phases still exist, but the diffraction peak intensity becomes strong, indicating the further growth and coarsening of the grains. Being distinct from the W5 HEA, no new phase transformation occurs in the W6-Co HEA after annealing at T_I_ and T_II_, indicating that the W6-Co HEA possesses a good thermal stability (Figure 3b). It suggests that the Co addition leads to the transformation from the metastable characteristic of W5 HEA to a more stable state in thermodynamics. However, the inset presents that the main diffraction peak of the W6-Co HEA shifts to the lower angle with the increased annealing temperature, suggesting a serious lattice distortion caused by the expansion of the lattice. Figure 3c reveals the formation of new phases of AlNi, AlGd, Gd-oxides, besides the primary BCC2 and FCC solid solutions for the as-annealed W6-Gd HEA at T_I_. The as-annealed products are unchanged at T_II_, except for the increased phase amounts of Gd-oxides. Moreover, compared with the W5 and W6-Co HEA, the highest T_m_ value of the W6-Gd HEA may be attributed to the high T_m_ values of precipitated intermetallic compounds of AlNi (1638 °C) and AlGd (1200 °C).

**Figure 4:**
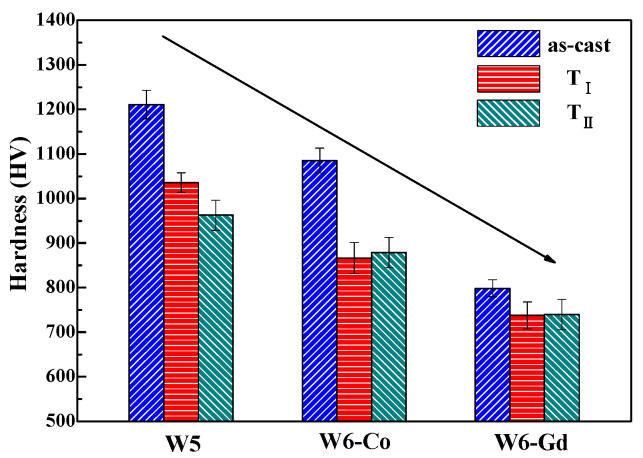
Vickers hardness of the as-cast and as-annealed W5, W6-Co, and W6-Gd HEAs.

Figure 4 shows the Vickers hardness (HV) of the as-cast and as-annealed HEAs. The W5 HEA displays the highest HV among the tested HEAs, and the as-cast W5 HEA possesses the highest hardness of 1210 HV. The additions of Co and Gd cause the decline of HV values, and the as-annealed W6-Gd HEA (T_II_) displays the maximal decline of HV (738). It suggest that the annealing treatment plays a negative effect on the HV of the as-cast samples, which is in agreement with Salishchec’s results [20]. With the increased annealing temperature, the internal stress of the as-cast HEAs gradually decreases as well as the microstructural coarsening. The effect of solid solution strengthening became the smaller, and strain softening was revealed in HEAs [21]. Compared with W6-Co and W6-Gd HEAs, the FeSi-rich phase, as the second strengthening phase in the W5 HEAs (especially the as-cast one) evenly distributes in the BCC solid solution matrix, which can contribute to the high HV values.

**Figure 5:**
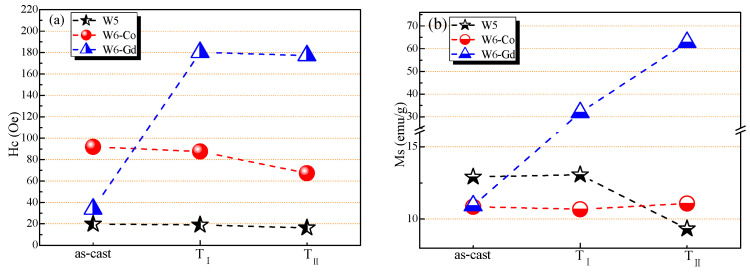

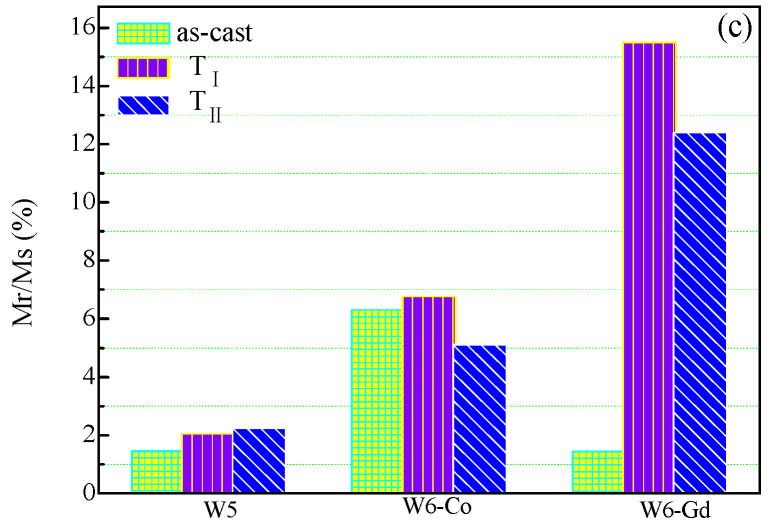
Magnetic properties of the as-cast and as-annealed W5, W6-Co, and W6-Gd HEAs: H_c_ (**a**), M_s_ (**b**) and M_r_/M_s_ (**c**).

The mass magnetization (M) as a function of the magnetic field intensity (H) for the as-cast and as-annealed samples was tested. The H_c_, M_s_, and M_r_/M_s_ of these HEAs are shown in Figure 5. All H_c_ values of the tested HEAs are in the range from 10 to 180 Oe (Figure 5a), indicating the soft magnetism nature of these HEAs. It suggests that the annealing treatment induces a weak decrease of H_c_ values for the W5 and W6-Co HEAs, but the H_c_ of W6-Gd HEA becomes large after annealing. The H_c_ is mainly affected by impurity, deformation, crystallite size, and stress, and the subsequent heat-treatment process [22]. Therefore, the H_c_ values of as-annealed W5 and W6-Co HEAs are slightly lower than those of the as-cast samples, suggesting that the former possess a little larger average crystallite size according to the well-known coercivity-crystal size relationship [8]. Moreover, the origin of the lower H_c_ can be attributed to the low number density of domain-wall pinning sites [23]. The as-annealed products of W6-Gd HEA contain complex phase compositions, and display the inhomogeneous characteristics, which obviously work towards the pinning effect of domain wall movement. Therefore the H_c_ values can be enhanced after the annealing treatment. 

The variations of M_s_ are exhibited in Figure 5b. In the as-cast state, there is no distinct difference in M_s_ for all the tested samples, and W5 HEA emerges with a slightly higher M_s_ of 12.91 emu/g. After annealing at T_I_, the M_s_ of W5 HEA remains nearly unchanged, whereas declines with a reduction of 27.7% at T_II_. There is no obvious magnetism changes revealed for the as-cast and as-annealed W6-Co HEAs, and the M_s_ values become stabilized at about 11 emu/g. This stability of M_s_ is resulted from the stable phase characteristic of W6-Co HEA in the annealing stage. Unlike W5 and W6-Co HEAs, the magnetism of W6-Gd HEA is enhanced during annealing treatment. The M_s_ value of the as-annealed W6-Gd HEA increased from 10.93 emu/g to 31.91 emu/g at T_I_, and further up to 62.78 emu/g at T_II_, suggesting increased soft magnetic properties.

From Figure 5c, the M_r_/M_s_ values of as-cast W5 and W6-Co HEAs are similar to their as-annealed states, which depend on their similar phase compositions. The as-annealed products of W6-Gd HEAs are significantly different from the as-cast one, and the M_r_/M_s_ values are enhanced from 1.44% (as-cast) to 15.5% (at T_I_). Moreover, the as-annealed W6-Gd HEAs reveal the highest M_r_/M_s_ values among the tested samples, indicating a better soft magnetism.

Residual stress exists in the as-cast HEAs which can deteriorate the soft magnetic properties. Appropriate heat treatment can induce stress relief, which is beneficial to improve the soft magnetic properties [24]. Therefore, except for the as-annealed W5 HEA at T_II_, the soft magnetic properties of the tested HEAs are properly improved by the structural relaxation through stress-relief annealing [25]. Notably, it suggests that M_s_ of the as-annealed W6-Gd HEA at T_II_ is about five times higher than that obtained for the as-cast one. Moreover, the magnetic properties are strongly dependent on the microstructure of the materials. The microstructure contribution to magnetism arises from morphology: properties such as magnetic anisotropy, magnetostriction, coercivity, and volume fraction of the precipitates. The decrease in M_s_ for as-annealed W5 at T_II_ can be related to the enhanced density of grain boundaries and the increase of volumetric fraction of BCC3 solid solutions around FeSi-rich phases, which reduce the magnetic moment. According to the effect of phase compositions on magnetic properties, the increase in M_s_ for the as-annealed W6-Gd HEA can be ascribed to the formation of Gd-oxides. Moreover, M_s_ is enhanced by increasing the contents of Gd-oxides after elevating the annealing temperature.

## 4. Conclusions

The phase composition, microstructures, microhardness, and magnetic properties of as-cast and as-annealed W5, W6-Co, and W6-Gd HEAs have been investigated. The as-cast and as-annealed W6-Co HEAs maintain the same phase compositions, and are composed of single FeSi-rich phases, indicating the stable phase characteristic. The addition of Gd obviously enhances T_m_ (1185 °C) compared with W5, owing to the exhibition of AlNi and AlGd with high melting points. As-cast W5 possesses the highest hardness of 1210 HV, which is attributed to the uniform distribution of the FeSi-rich phase in the matrix. All the tested HEAs display soft magnetic properties. Moreover, the M_s_ and M_r_/M_s_ values of W6-Gd were enhanced from 10.93 emu/g to 62.78 emu/g and from 1.44% to 15.50% via the annealing process, respectively. It suggests that Gd-oxides are beneficial to the enhancement of magnetic properties in W6-Gd. 

## Figures and Tables

**Table 1:** Phase products of as-cast and as-annealed W5, W6-Co, and W6-Gd high entropy alloys (HEAs) at T_I_ and T_II_, identified distinctly from XRD patterns.

HEAs	As-Cast	As-Annealed	
		T_I_	T_II_
W5	BCC1+FeSi-rich	BCC3+FeSi-rich	BCC3+FeSi-rich
W6-Co	FeSi-rich	FeSi-rich	FeSi-rich
W6-Gd	BCC2+FCC	BCC2 + FCC + AlNi + AlGd + Gd-oxide	BCC2 + FCC + AlNi + AlGd + Gd-oxide

**Table 2:** Chemical compositions of representative regions for W5, W6-Co, and W6-Gd HEAs obtained by energy dispersive spectrometry (EDS).

Regions	Elements (at. %)						
	Fe	Si	B	Al	Ni	Co	Gd
A	11.36	10.01	-	36.29	42.34	-	-
B	39.6	33.8	-	12.95	13.65	-	-
C	80.9	1.65	10.97	2.6	3.87	-	-
D	41.15	27.85	-	6.38	10.49	14.13	-
E	11.42	12.51	-	17.59	34.78	23.7	
F	24.72	22.07	-	2.1	27.66	23.45	
G	24.5	27.9	-	0.48	17.41	-	29.71
H	14.57	2.55	-	51.32	11.06	-	20.5

## References

[1] Yeh J.W., Chen S.K., Lin S.J., Gan J.Y., Chin T.S., Shun T.T., Tsau C.H., Chang S.Y. (2004). Nanostructured High-Entropy Alloys with Multiple Principal Elements: Novel Alloy Design Concepts and Outcomes. Adv. Eng. Mater..

[2] Cantor B., Chang I.T.H., Knight P., Vincent A.J.B. (2004). Microstructure development in equiatomic multicomponent alloys. Mater. Sci. Eng. A.

[3] Zhang Y., Zuo T.T., Tang Z., Gao M.C., Dahmen K.A., Liaw P.K., Lu Z.P. (2014). Microstructures and properties of high-entropy alloys. Prog. Mater. Sci..

[4] Dong Y., Zhou K.Y., Lu Y.P., Gao X.X., Wang T.M., Li T.J. (2014). Effect of Vanadium Addition on the Microstructure and Properties of AlCoCrFeNi High Entropy Alloy. Mater. Des..

[5] Wu Z., Bei H., Pharr G.M., George E.P. (2014). Temperature dependence of the mechanical properties of equiatomic solid solution alloys with face-centered cubic crystal structures. Acta Mater..

[6] Chen Y.Y., Duval T., Hung U.D., Yeh J.W., Shih H.C. (2005). Microstructure and electrochemical properties of high entropy alloys-a comparison with type-304 stainless steel. Corros. Sci..

[7] Ji W., Wang W.M., Wang H., Zhang J.Y., Wang Y.C., Zhang F., Fu Z.Y. (2015). Alloying behavior and novel properties of CoCrFeNiMn high-entropy alloy fabricated by mechanical alloying and spark plasma sintering. Intermetallics.

[8] Li P.P., Wang A.D., Liu C.T. (2017). A ductile high entropy alloy with attractive magnetic properties. J. Alloys Compd..

[9] Zuo T.T., Gao M.C., Ouyang L.Z., Yang X., Cheng Y.Q., Feng R., Chen S.Y., Liaw P.K., Hawk J.A., Zhang Y. (2017). Tailoring magnetic behavior of CoFeMnNiX (X = Al, Cr, Ga, and Sn) high entropy alloys by metal doping. Acta Mater..

[10] He J.Y., Liu W.H., Wang H., Wu Y., Liu X.J., Nieh T.G., Lu Z.P. (2014). Effects of Al addition on structural evolution and tensile properties of the FeCoNiCrMn high-entropy alloy system. Acta Mater..

[11] Xu J., Axinte E., Zhao Z., Wang Y. (2016). Effect of C and Ce addition on the microstructure and magnetic property of the mechanically alloyed FeSiBAlNi high entropy alloys. J. Magn. Magn. Mater..

[12] Zhuang Y.X., Xue H.D., Chen Z.Y., Hu Z.Y., He J.C. (2013). Effect of annealing treatment on microstructures and mechanical properties of FeCoNiCuAl high entropy alloys. Mater. Sci. Eng. A.

[13] Zhu X., Zhou X., Yu S., Wei C., Xu J., Wang Y. (2017). Effects of annealing on the microstructure and magnetic property of the mechanically alloyed FeSiBAlNiM (M = Co, Cu, Ag) amorphous high entropy alloys. J. Magn. Magn. Mater..

[14] Wang J., Zheng Z., Xu J., Wang Y. (2014). Microstructure and magnetic properties of mechanically alloyed FeSiBAlNi (Nb) high entropy alloys. J. Magn. Magn. Mater..

[15] Zhang Q., Xu H., Tan X.H., Hou X.L., Wu S.W., Tan G.S., Yu L.Y. (2017). The effects of phase constitution on magnetic and mechanical properties of FeCoNi(CuAl)_x_ (x = 0–1.2) high-entropy alloys. J. Alloys Compd..

[16] Xu J., Shang C., Ge W., Jia H., Liaw P.K., Wang Y. (2016). Effects of elemental addition on the microstructure, thermal stability, and magnetic properties of the mechanically alloyed FeSiBAlNi high entropy alloys. Adv. Powder Technol..

[17] Tsai M.H., Yuan H., Cheng G., Xu W., Tsai K.Y., Tsai C.W., Jian W.W., Juan C.C., Shen W.J., Chuang M.H. (2013). Morphology, structure and composition of precipitates in Al_0.3_CoCrCu_0.5_FeNi high-entropy alloy. Intermetallics.

[18] Chen C., Pang S., Cheng Y., Zhang T. (2016). Microstructure and mechanical properties of Al_20−x_Cr_20+0.5x_Fe_20_Co_20_Ni_20+0.5x_ high entropy alloys. J. Alloys Compd..

[19] Zhang Y., Zuo T., Cheng Y., Liaw P.K. (2013). High-entropy alloys with high saturation magnetization, electrical resistivity, and malleability. Sci. Rep..

[20] Salishchev G.A., Tikhonovsky M.A., Shaysultanov D.G., Stepanov N.D., Kuznetsov A.V., Kolodiy I.V., Tortika A.S., Senkov O.N. (2014). Effect of Mn and V on structure and mechanical properties of high-entropy alloys based on CoCrFeNi system. J. Alloys Compd..

[21] Zhu Z.G., Ma K.H., Yang X., Shek C.H. (2017). Annealing effect on the phase stability and mechanical properties of (FeNiCrMn)_(100−x)_Co_x_ high entropy alloys. J. Alloys Compd..

[22] Sun G.F., Qiang W.J. (2007). Magnetic Material.

[23] Bitoh T., Makino A., Inoue A. (2006). Origin of low coercivity of (Fe_0.75_B_0.15_Si_0.10_)_100−x_Nb_x_(x = 1–4) glassy alloys. J. Appl. Phys..

[24] Wei R., Tao J., Sun H., Chen C., Sun G.W., Li F.S. (2017). Soft magnetic Fe_26.7_Co_26.7_Ni_26.6_Si_9_B_11_ high entropy metallic glass with good bending ductility. Mater. Lett..

[25] Kong F.L., Chang C.T., Inoue A., Shalaan E., Al-Marzouki F. (2014). Fe-based amorphous soft magnetic alloys with high saturation magnetization and good bending ductility. J. Alloys Compd..

